# Modified the 8th AJCC staging system for patients with advanced prostate cancer: a study based on SEER database

**DOI:** 10.1186/s12894-022-01135-z

**Published:** 2022-11-16

**Authors:** Chengwen Sun, Dongrong Yang, Jin Zhu, Yibin Zhou, Congming Xiang, Sheng Wu

**Affiliations:** 1 Department of Urology, The Second Affiliated Hospital of Soochow University, No.1055 Sanxiang Road, 215000 Suzhou, China; 2 Department of Urology, The Affiliated Hospital of Jiangnan University, 214000 Wuxi, China

**Keywords:** Prostate cancer, American Joint Committee on Cancer 8th, Modified stage, prognosis

## Abstract

**Background:**

The American Joint Committee on Cancer (AJCC) 8th staging system of prostate cancer may be insufficient in predicting the prognosis of some staged patients. This study aimed to modify the AJCC 8th staging system in patients with advanced prostate cancer.

**Methods:**

Data of patients with advanced prostate cancer from the Surveillance, Epidemiology, and End Results (SEER) database between 2004 and 2016 were enrolled in this cohort study. All patients were divided into the training set and the testing set with a ratio of 6:4. Multivariate Cox survival model was utilized to obtain the nomogram score for each stage variable. The modified staging system was based on the total nomogram score. The C-index and Kaplan-Meier (K-M) curves were used to show the prognostic prediction effect of patients with different staging systems.

**Results:**

A total of 28,006 patients were included for analysis. T stage, N stage, M stage, primary Gleason pattern score, secondary Gleason pattern score, and PSA level were included as stage variables. Patients with AJCC stage III C [hazard ratio (HR) = 4.17, 95% confidence interval (CI), 3.39–5.13] and AJCC stage IV B (HR = 3.19, 95%CI, 1.79–5.69) were associated with worse prognosis compared with those of AJCC stage III B, while no statistical significance was found in patients with stage IV A (*P* > 0.05). In terms of the modified staging system, patients with modified stage III C (HR = 2.06, 95%CI, 1.46–2.92), modified stage IV A (HR = 6.91, 95%CI, 4.81–9.94), and modified stage IV B (HR = 21.89, 95%CI, 14.76–32.46) were associated with a poorer prognosis compared with patients with modified stage III B. The prognostic ability (C-index) of the modified staging system (0.789; 95%CI, 0.777–0.801) was better than that of the AJCC 8th edition system (0.762; 95%CI, 0.748–0.776) (0.789 vs. 0.762, *P* = 0.004). The K-M curves indicated that the modified staging system may be distinguished prognostic differences in patients with different stages.

**Conclusion:**

Modified staging system may be better than AJCC 8th staging system for predicting prognosis in prostate cancer patients. The AJCC 8th staging system should be further optimized.

## Background

Prostate cancer is the most common cancer in men worldwide after lung cancer [1]. In 2020, approximately1.4 million men were newly diagnosed with prostate cancer and 375,304 men died from this disease worldwide [1]. Staging according to the patient’s disease status is an indispensable indicator for patient treatment and prognosis prediction [2, 3].

The American Joint Committee on Cancer (AJCC) groups patients according to the stage of disease and has been used to estimate patient prognosis [4]. The classification of tumors in the AJCC staging system is based on the local extent of the primary tumor (T stage), as well as spread to lymph nodes (N stage) and distant metastases (M stage). For the staging of prostate cancer, the Gleason grade and prostate-specific antigen (PSA) levels were incorporated into the AJCC staging system in 2002 (6th edition) and 2010 (7th edition), respectively [5, 6]. The AJCC 8th edition of the prostate cancer staging system, recently updated in 2016 [7], undoubtedly has advantages over previous systems in staging and predicting patient prognosis [8, 9]. However, the AJCC 8th edition staging system may be insufficient in predicting the prognosis of some staged patients. For example, prostate cancer patients with higher PSA levels or higher tumor grades may have a worse prognosis than the patients with higher stage but lower PSA level or lower tumor grade [10]. Several studies also indicated that the higher-grade group has a significantly lower prognosis than the lower-grade group and should be considered a distinct group [8, 11–13]. Therefore, we attempted to modify the AJCC 8th edition prostate cancer staging system to match patients with higher stages with poorer prognoses.

Early-stage patients are commonly diagnosed with localized, low-risk prostate cancer with excellent treatment outcomes [14–16]. In this study, we aimed to modify the staging of only advanced prostate cancer. Comparisons of the AJCC 8th edition staging system and the modified staging system for prognosis prediction in patients with advanced prostate cancer were analyzed.

## Methods

### Data sources and patients

Data of primary prostate cancer patients from the Surveillance, Epidemiology, and End Results (SEER 18 registry) database between 2004 and 2016 were utilized for the analysis of this cohort study [17]. Science the reliable prostatic specific antigen (PSA) data were not available before 2004, this study only analyzed the data after 2004. Patients with prostate cancer were identified by the International Classification of Diseases for Oncology, Third Edition (ICD-O-3) histology code (C61.9). Patients diagnosed with primary prostate cancer were enrolled in the study. Patients were excluded according to the following criteria: (1) missing data of TNM stage, Gleason score, and PSA level; (2) patients with survival months of 0 or lost to follow-up; (3) patients with T1 or T2 stage and Gleason score < 8 (excluded AJCC 8th edition stage I to stage III A). The survival status of patients was determined based on the death registration system. The follow-up was from the time the patient was diagnosed with prostate cancer until the patient died during the study period or until the publication of the database. This study was based on de-identified data from a publicly available database and did not involve interaction with human subjects. Therefore, this study did not require approval from the Institutional Review Board of The Second Affiliated Hospital of Soochow University.

## Variables

Information of patients including age, race (white, black, others, and unknown), marital status (divorced, married, separated, single, unmarried or domestic partner, widowed, and unknown), insurance status (any Medicaid, insured, insured/no specifics, uninsured, and unknown), histology (adenocarcinoma and non-adenocarcinoma), stage (distant site/node involved, localized only, regional by direct extension and lymph nodes, regional by direct extension only, and regional lymph nodes involved only), tumor size, radiotherapy (yes and no/unknown), surgery (yes and no/unknown), chemotherapy (yes and no/unknown), T stage (T3a, T3b, and T4), N stage (N0 and N1), M stage (M0 and M1), AJCC stage 8th edition (III B, III C, IV A, and IV B), PSA levels (mg/ml), primary Gleason pattern scores, secondary Gleason pattern scores, and survival months were collected. Reconstruction of the 8th edition AJCC stage for each patient was based on PSA, Gleason grade/grade group and 6th edition TNM stage.

## Procedures

Staging variables such as TNM stage, PSA levels, primary Gleason pattern scores, and secondary Gleason pattern scores were included in a multivariate Cox survival model to obtain the nomogram score for each variable. According to the total nomogram score, all patients were reclassified into four stages (modified III B stage, modified III C stage, modified IV A stage, and modified IV B stage). A comparison of the survival prediction between the modified staging system and the AJCC 8th edition staging system was performed. The C-index was used to evaluate the predictive performance, and the DeLong test was utilized for the comparison between the two staging systems. Furthermore, the Kaplan-Meier (K-M) curves were used to show the survival of patients with different staging systems.

### Statistical analysis

Quantitative data were tested for normality by the Kolmogorov-Smirnov test. Quantitative data with normal distribution were expressed as mean ± standard deviation (SD), and independent samples t-test was used for comparison between two groups. Quantitative data that were non-normal distribution were described as a median and interquartile range [M (Q1, Q3)], and the comparison between groups was conducted using the Mann-Whitney U rank-sum test. Enumeration data were presented as numbers and constituent ratio [n (%)], and the chi-square test was utilized for comparison between groups.

Data were randomly divided into the training set and testing set with a ratio of 6:4. Difference analysis was conducted between dead patients and alive patients. Univariable and multivariable analyses were performed to assess the impact of TNM stage, PSA levels, primary Gleason pattern scores, and secondary Gleason pattern scores on the prognosis of patients. In the analysis of the relationship between AJCC 8th staging system and modified staging system and prognosis of patients, three models were established: (1) model 1 was univariable analysis model; (2) model 2 was multivariable analysis model that adjusted for age, race, marital status, and insurance status; (3) model 3 was also multivariable analysis model that adjusted for age, race, marital status, insurance status, and stage. Statistical analyses were two-sided tests, and *P* < 0.05 was considered statistically significant. Univariable and multivariable analyses were performed by SAS 9.04 software (SAS Institute Inc., Cary, NC, USA), and the other analyses were performed by R 3.6.3 software.

## Results

### Characteristics of patients

**Fig. 1:**
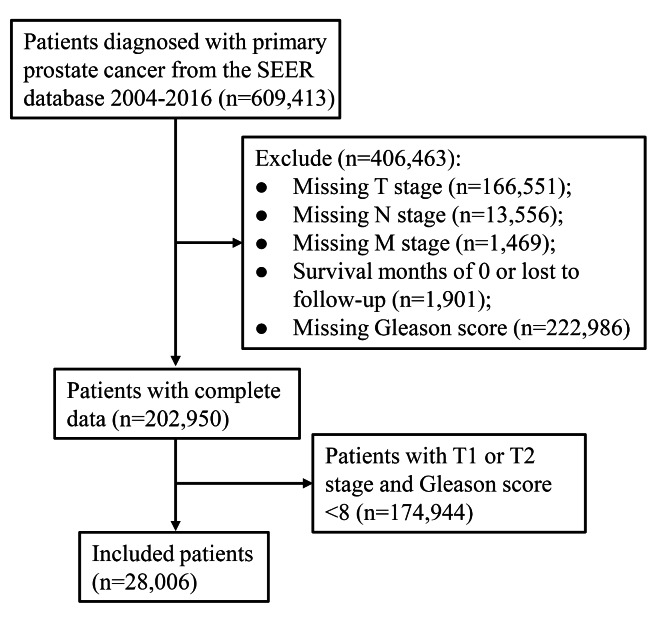
Flow chart of the study population

From 2004 to 2016, 609,413 patients with primary prostate cancer were extracted, 406,463 patients with incomplete data were excluded, 174,944 patients with T1 or T2 stage and Gleason score < 8 were also excluded, and a total of 28,006 patients were included in this study (Fig. 1). Of these 28,006 patients, 16,803 patients were in the training set and 11,203 patients were in the testing set. Table 1 presents the characteristics of patients in the training set. The mean age of these patients was 63.66 ± 7.80 years and the median tumor size was 114.00 (33.00, 114.00) mm. In terms of AJCC 8th edition, 13,307 (79.19%) patients were III B stage, 520 (3.09%) patients were.

**Table 1:** Characteristics of patients with advanced prostate cancer in the training set

Variables	Total (n = 16,803)	Alive (n = 15,299)	Dead (n = 1504)	Statistics	*P*
Age (years), mean ± SD	63.66 ± 7.80	63.19 ± 7.38	68.45 ± 10.07	t=-19.75	< 0.001
Race, n (%)				χ^2^ = 21.517	< 0.001
Blacks	2222 (13.22)	1977 (12.92)	245 (16.29)		
Other (American Indian/AK Native, Asia	1065 (6.34)	972 (6.35)	93 (6.18)		
Unknown	166 (0.99)	162 (1.06)	4 (0.27)		
Whites	13,350 (79.45)	12,188 (79.67)	1162 (77.26)		
Marital status, n (%)				χ^2^ = 133.345	< 0.001
Divorced	1246 (7.42)	1093 (7.14)	153 (10.17)		
Married	11,986 (71.33)	11,077 (72.40)	909 (60.44)		
Separated	142 (0.85)	120 (0.78)	22 (1.46)		
Single	1937 (11.53)	1716 (11.22)	221 (14.69)		
Unknown	997 (5.93)	902 (5.90)	95 (6.32)		
Unmarried or Domestic Partner	49 (0.29)	43 (0.28)	6 (0.40)		
Widowed	446 (2.65)	348 (2.27)	98 (6.52)		
Insurance status, n (%)				χ^2^ = 106.765	< 0.001
Any Medicaid	1006 (5.99)	837 (5.47)	169 (11.24)		
Insurance status unknown	358 (2.13)	318 (2.08)	40 (2.66)		
Insured	12,589 (74.92)	11,597 (75.80)	992 (65.96)		
Insured/No specifics	2543 (15.13)	2268 (14.82)	275 (18.28)		
Uninsured	307 (1.83)	279 (1.82)	28 (1.86)		
Histology, n (%)				χ^2^ = 43.039	< 0.001
adenocarcinoma	205 (1.22)	160 (1.05)	45 (2.99)		
Non-adenocarcinoma	16,598 (98.78)	15,139 (98.95)	1459 (97.01)		
Stage, n (%)				χ^2^ = 1722.360	< 0.001
Distant site/node involved	1143 (6.80)	537 (3.51)	606 (40.29)		
Localized only	12 (0.07)	12 (0.08)	0 (0.00)		
Regional by direct extension and lymph nodes	1868 (11.12)	1689 (11.04)	179 (11.90)		
Regional by direct extension only	13,778 (82.00)	13,059 (85.36)	719 (47.81)		
Regional by direct extension only	2 (0.01)	2 (0.01)	0 (0.00)		
Tumor size (mm), M (Q_1_, Q_3_)	114.00 (33.00, 114.00)	114.00 (31.00, 114.00)	114.00 (114.00, 114.00)	Z = 14.757	< 0.001
Radiotherapy, n (%)				χ^2^ = 8.408	0.004
No/Unknown	14,178 (84.38)	12,870 (84.12)	1308 (86.97)		
Yes	2625 (15.62)	2429 (15.88)	196 (13.03)		
Surgery, n (%)				χ^2^ = 1588.610	< 0.001
No/Unknown	2345 (13.96)	1624 (10.62)	721 (47.94)		
Surgery performed	14,458 (86.04)	13,675 (89.38)	783 (52.06)		
Chemotherapy, n (%)				χ^2^ = 214.788	< 0.001
No/unknown	16,530 (98.38)	15,119 (98.82)	1411 (93.82)		
Yes	273 (1.62)	180 (1.18)	93 (6.18)		
T stage, n (%)				χ^2^ = 1067.498	< 0.001
T3a	9258 (55.10)	8837 (57.76)	421 (27.99)		
T3b	4976 (29.61)	4544 (29.70)	432 (28.72)		
T4	2569 (15.29)	1918 (12.54)	651 (43.28)		
N stage, n (%)				χ^2^ = 460.789	< 0.001
N0	14,343 (85.36)	13,340 (87.20)	1003 (66.69)		
N1	2460 (14.64)	1959 (12.80)	501 (33.31)		
M stage, n (%)				χ^2^ = 2881.475	< 0.001
M0	15,709 (93.49)	14,793 (96.69)	916 (60.90)		
M1	1094 (6.51)	506 (3.31)	588 (39.10)		
AJCC 8th, n (%)				χ^2^ = 3090.351	< 0.001
IIIB	13,307 (79.19)	12,686 (82.92)	621 (41.29)		
IIIC	520 (3.09)	410 (2.68)	110 (7.31)		
IVA	1882 (11.20)	1697 (11.09)	185 (12.30)		
IVB	1094 (6.51)	506 (3.31)	588 (39.10)		
PSA (mg/ml), M (Q_1_, Q_3_)	0.09 (0.06, 0.18)	0.08 (0.05, 0.16)	0.22 (0.08, 0.98)	Z = 24.425	< 0.001
Primary Gleason pattern scores, mean ± SD	3.63 ± 0.61	3.58 ± 0.59	4.07 ± 0.66	t=-27.63	< 0.001
Secondary Gleason pattern scores, mean ± SD	3.84 ± 0.70	3.80 ± 0.68	4.21 ± 0.73	t=-21.29	< 0.001
Survival months, M (Q_1_, Q_3_)	42.00 (23.00, 63.00)	44.00 (25.00, 64.00)	26.00 (14.00, 42.00)	Z=-25.322	< 0.001

Note: AJCC, American Joint Committee on Cancer; PSA, prostate-specific antigen

III C stage, 1,882 (11.20%) patients were IV A stage, and 1,094 (6.51%) patients were IV B stage. The median PSA levels of patients was 0.09 (0.06, 0.18) mg/ml. The mean.

primary Gleason pattern scores and secondary Gleason pattern scores were 3.63 ± 0.61 and 3.84 ± 0.70, respectively. The median survival month of patients was 42.00 (23.00, 63.00) months. The median follow-up time was 42 months and the follow-up time ranged from 1 month to 83 months. At the end of follow-up, 152,99 (91.05%) patients were alive and 1504 (8.95%) patients had died.

Difference analysis between alive patients and dead patients demonstrated that significant differences were observed between the two groups of patients in age, race, marital status, insurance status, histology, stage, tumor size, chemotherapy, T stage, N stage, M stage AJCC stage, PAS levels, primary Gleason pattern scores, secondary Gleason pattern scores, and survival months (all *P* < 0.05, Table 1).

## Development of a modified staging system

Table 2 presents the impact of TNM stage, Gleason scores, and PSA levels on the survival of patients with primary prostate cancer. These factors were included in a multivariate Cox survival model to obtain the nomogram score for each variable. Table 3 shows the nomogram scores for specific numerical values of each factor. The nomogram score ranges for each factor were as follows: T stage (0–37 scores), N stage (0–4 scores), M stage (0–67 scores), primary Gleason pattern score (0-100 scores), secondary Gleason pattern score (0–58 scores), and PSA level (0–26 scores). Patients were divided into four stages based on the total nomogram score: modified stage III B (0–75 scores), modified stage III C (75–150 scores), modified stage IV A (150–225 scores), and modified stage IV B (225–292 scores).

**Table 2:** Impact of TNM stage, Gleason scores, and PSA levels on the survival of patients with advanced prostate cancer

Variables	Univariable		Multivariable	
	HR (95%CI)	P	HR (95%CI)	P
T stage				
T3a	Ref			
T3b	2.00 (1.75–2.29)	< 0.001	1.33 (1.16–1.53)	< 0.001
T4	7.01 (6.26-8.00)	< 0.001	2.44 (2.11–2.82)	< 0.001
N stage				
N0	Ref			
N1	3.60 (3.23–4.01)	< 0.001	1.09 (0.97–1.23)	0.153
M stage				
M0	Ref			
M1	17.49 (15.73–19.46)	< 0.001	5.08 (4.39–5.87)	< 0.001
Primary Gleason pattern scores	3.61 (3.33–3.91)	< 0.001	1.83 (1.68-2.00)	< 0.001
Secondary Gleason pattern scores	2.46 (2.28–2.65)	< 0.001	1.42 (1.32–1.54)	< 0.001
PSA levels	7.64 (6.76–8.64)	< 0.001	1.90 (1.62–2.22)	< 0.001

Note: HR, hazard ratio; CI, confidence interval; PSA, prostate-specific antigen; Multivariable analysis adjusted for 5 other variables except the analysis variable

**Table 3:** The nomogram scores for specific numerical values of each stage variable

Stage variables	scores
T stage	T3a – 0 T3b – 12 T4–37
M stage	M0–0 M1–67
N stage	N0–0 N1–4
Primary Gleason pattern scores	1–0 2–25 3–35 4–75 5–100
Secondary Gleason pattern scores	1–0 2–15 3–29 4–44 5–58
PSA levels (mg/ml)	0–0 0.1–3 0.2–5 0.4–11 0.5–13 0.6–16 0.7–18 0.8–21 0.9–24 1–26

## Comparison of the prognostic performance between modified staging and AJCC staging

Table 4 demonstrates the association of modified staging system and AJCC staging system with survival in patients with prostate cancer in the training set and testing set. In the training set, patients with modified stage III C (HR = 2,32, 95%CI, 1.64–3.28), modified stage IV A (HR = 11.35, 95%CI, 8.00-16.10), and modified stage IV B (HR = 65.89, 95%CI, 46.45–93.48) were associated with a poorer prognosis compared with patients with modified stage III B. After adjusting for age, race, marital status, insurance status, and stage, Patients with modified stage III C (HR = 2.06, 95%CI, 1.46–2.92), modified stage IV A (HR = 6.91, 95%CI, 4.81–9.94), and modified stage IV B (HR = 21.89, 95%CI, 14.76–32.46) were still related to a poorer prognosis. In terms of the AJCC staging system, compared with stage III B, patients with stage III C (HR = 5.34, 95%CI, 4.36–6.54), stage IV A (HR = 2.44, 95%CI, 2.07–2.88), and stage IV B (HR = 22,49, 95%CI, 20.04–25.25) had poorer prognosis. After adjusting for the above variables, patients with stage III C (HR = 4.17, 95%CI, 3.39–5.13) and stage IV B (HR = 3.19, 95%CI, 1.79–5.69) were associated with worse prognosis, while no statistical significance was found in patients with stage IV A (*P* > 0.05). Similar results were also observed in the testing set. The prognostic ability (C-index) of the modified staging and AJCC 8th edition systems in the training set was 0.789 (95%CI, 0.777–0.801) and 0.762 (95%CI, 0.748–0.776), respectively (0.789 vs. 0.762, Z = 2.870, *P* = 0.004). In the testing set, the prognostic ability of the modified staging was also better than that of the AJCC 8th edition system [C-index, 0.794 (95%CI, 0.778–0.810) vs. 0.749 (95%CI, 0.731–0.767), Z = 3.662, *P* < 0.001].

**Table 4:** Association of modified staging system and AJCC staging system with survival in patients with prostate cancer in the training set and testing set

	Model 1		Model 2		Model 3	
	h (95%CI)	P	HR (95%CI)	P	HR (95%CI)	P
**Training set**						
Modified stage						
III B	Ref					
III C	2.32 (1.64–3.28)	< 0.001	2.03 (1.43–2.87)	< 0.001	2.06 (1.46–2.92)	< 0.001
IV A	11.35 (8.00-16.10)	< 0.001	8.56 (6.03–12.16)	< 0.001	6.91 (4.81–9.94)	< 0.001
IV B	65.89 (46.45–93.48)	< 0.001	42.71 (29.98–60.85)	< 0.001	21.89 (14.76–32.46)	< 0.001
AJCC 8th stage						
III B	Ref					
III C	5.34 (4.36–6.54)	< 0.001	4.31 (3.51–5.29)	< 0.001	4.17 (3.39–5.13)	< 0.001
IV A	2.44 (2.07–2.88)	< 0.001	2.42 (2.05–2.85)	< 0.001	0.64 (0.35–1.15)	0.132
IV B	22.49 (20.04–25.25)	< 0.001	16.42 (14.52–18.56)	< 0.001	3.19 (1.79–5.69)	< 0.001
**Testing set**						
Modified stage						
III B	Ref					
III C	2.34 (1.52–3.60)	< 0.001	2.10 (1.36–3.23)	< 0.001	2.18 (1.41–3.35)	< 0.001
IV A	13.96 (9.06–21.50)	< 0.001	10.55 (6.83–16.30)	< 0.001	9.17 (5.85–14.37)	< 0.001
IV B	66.18 (42.87-102.17)	< 0.001	43.81 (28.22–68.01)	< 0.001	25.83 (15.85–42.08)	< 0.001
AJCC 8th stage						
III B						
III C	4.43 (3.43–5.71)	< 0.001	3.48 (2.69–4.50)	< 0.001	3.21 (2.47–4.17)	< 0.001
IV A	2.75 (2.27–3.33)	< 0.001	2.74 (2.26–3.31)	< 0.001	1.82 (0.74–4.49)	0.194
IV B	20.41 (17.70-23.52)	< 0.001	14.39 (12.37–16.73)	< 0.001	6.92 (2.83–16.87)	< 0.001

Note: HR, hazard ratio; CI, confidence interval; model 1, univariable analysis; model 2, multivariable analysis model that adjusted for age, race, marital status, and insurance status; model 3, multivariable analysis model that adjusted for age, race, marital status, insurance status, and stage

**Fig. 2:**
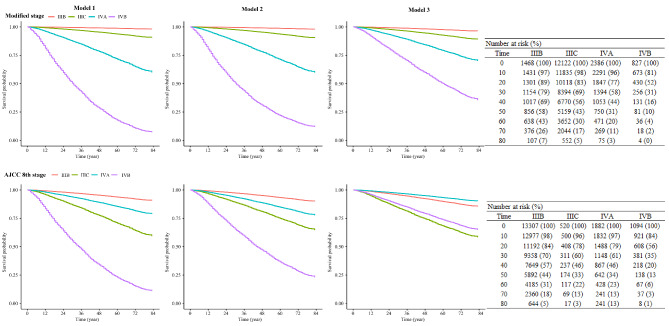
Kaplan-Meier (K-M) survival curves for prostate cancer patients using the modified staging and AJCC 8th edition staging system. model 1, univariable analysis; model 2, multivariable analysis model that adjusted for age, race, marital status, and insurance status; model 3, multivariable analysis model that adjusted for age, race, marital status, insurance status, and stage

K-M survival curves for prostate cancer patients using the modified staging and AJCC 8th edition staging system were presented in Fig. 2. Before adjusting for variables, both the modified staging system and the AJCC 8th edition staging system displayed good discriminative ability for the prognosis of patients with different stages of prostate cancer. In the modified staging system, the higher patient stage corresponds to a worse prognosis, while stage IV A patients have a better prognosis than stage III C patients in the AJCC 8th edition staging system. After adjusting for all variables, higher patient stages were still associated with worse prognosis in the modified staging system, while the AJCC 8th staging system may not be able to differentiate the overall survival of patients with AJCC stage III B and stage IV A, and patients with AJCC stage III C and stage IV B very well.

## Discussion

The AJCC 8th edition staging system was utilized to estimate the prognosis of cancer patients [4]. However, the AJCC 8th edition staging system may not be effective in distinguishing the prognosis of different advanced prostate cancer patients. This study modified the AJCC staging of patients with advanced prostate cancer based on their TNM stage, Gleason score, and PSA level. Our results indicated that there was no significant difference in the survival of patients with AJCC stage III C, stage IV A, and stage IV B, while the modified staging system may be distinguished prognostic difference in patients with different stages. Furthermore, the prognostic predictive ability of the modified staging for advanced prostate cancer may be better than that of the AJCC 8th edition systems.

TNM stage, Gleason score, and PSA level are the basis for AJCC staging in prostate cancer patients [18, 19]. Detailed subgroup classification of prostate cancer patients in the 8th edition of the AJCC staging system has been updated in 2016 [20]. Previous studies have found that the prognosis of patients with Gleason score 3 + 4 and 4 + 3 is significantly different and cannot be considered as the same prognosis group [11, 21, 22]. In the 8th edition AJCC staging system of prostate cancer, the Gleason score was updated, specifically, the scores 3 + 4 and 4 + 3 were divided into grade group 2 and grade group 3, respectively [23]. In our modified staging system, the primary Gleason pattern scores and secondary Gleason pattern scores were independently incorporated into the system. Furthermore, the nomogram score corresponding to the specific value of each variable was obtained by multivariate analysis of the influence of TNM stage, Gleason score, and PSA level on the prognosis of patients. The modified staging system was constructed based on the patient’s total nomogram score. In our modified staging system, the effect of each variable is in a different state on patient prognosis was considered.

Several studies have evaluated the predictive effect of the AJCC 8th edition staging system on prognosis in patients with prostate cancer [9, 10, 24]. The AJCC 8th edition staging system has updated the 7th edition staging system based on the TNM stage and Gleason score [23]. A previous study indicated that the prognostic predictive ability of the AJCC 8th edition staging system is better than that of the AJCC 7th staging system [9]. However, the AJCC 8th staging system may have some stages that cannot be matched with the prognosis of prostate cancer patients, that is, the higher stage did not necessarily correspond to a worse prognosis. Xiao et al. found that prostate cancer patients with higher PSA levels or higher tumor grades may have a worse prognosis than patients with the higher stage but lower PSA level or lower tumor grade [10]. Numerous studies have reached similar results that the higher-grade group should be considered a distinct group due to its significantly worse prognosis than the lower-grade group [8, 11–13]. Our results also supported that the patients with stage IV A may have a better prognosis than patients with stage III C in the AJCC 8th edition staging system. Therefore, in our modified staging system T stage, N stage, M stage, primary Gleason pattern scores, secondary Gleason pattern scores, and PSA level were included as independent variables. The staging of patients was based on a total score formed from the nomogram scores contributed by each variable. The C index of the modified staging system (0.789) was higher than that of the AJCC 8th edition staging system (0.762). Furthermore, the stage of patients was matched with prognosis, that is, patients with high stage were associated with poorer prognosis. Our modified staging system may provide a reference for further optimization of the AJCC 8th edition staging system.

We have modified the staging of advanced patients in the AJCC 8th edition staging system of prostate cancer. The modified staging system was based on nomogram scores of variables that affect a patient’s prognosis, such as TNM stage, Gleason score, and PSA level. Modified staging system demonstrated superior ability to predict prognosis in prostate cancer patients over AJCC 8th staging system. However, some limitations of the current study should be considered. First, this study was based on the data cohort of the SEER database and lacks an independent external data cohort for validation. Second, the PSA data before 2004 does not exist in the SEER database, which may affect data integrity. Third, some detailed treatment and recurrence information such as hormone therapy, the number of lymph nodes removed, and nerve sparing were not recorded in the SEER database, and we also did not consider the effect of surgical margin status, which may affect the prediction of patient prognosis.

## Conclusion

The staging of advanced patients in the AJCC 8th edition staging system for prostate cancer was modified. The modified staging system was based on the total nomogram score of the T stage, N stage, M stage, primary Gleason pattern score, secondary Gleason pattern score, and PSA level. The prognostic ability of the modified staging system was better than that of the AJCC 8th edition system. Furthermore, the modified staging system may be distinguished prognostic differences in patients with different stages compared with the AJCC 8th edition system, allowing for a more accurate assessment of the prognosis of patients with prostate cancer. The AJCC 8th staging system should be further optimized.

## Data Availability

The datasets generated and/or analyzed during the current study are available in the SEER database, https://seer.cancer.gov/. (accession number: 12,489-Nov2021).

## Abbreviations

AJCC: American Joint Committee on Cancer
PSA: prostate-specific antigen
